# Assessment of Antimicrobial Effects on Broiler Gut Barrier Through Histopathology and Immunohistochemistry of Tight-Junction Proteins

**DOI:** 10.3389/fvets.2022.830073

**Published:** 2022-03-29

**Authors:** Matteo Cuccato, Frine Eleonora Scaglione, Cinzia Centelleghe, Sara Divari, Bartolomeo Biolatti, Paola Pregel, Francesca Tiziana Cannizzo

**Affiliations:** ^1^Department of Veterinary Science, University of Turin, Turin, Italy; ^2^Department of Comparative Biomedicine and Food Science, University of Padua, Padua, Italy

**Keywords:** antimicrobial, broiler, intestine, gut barrier, tight-junction, coccidiostat

## Abstract

In recent years, antimicrobial (AM) use in poultry farming has been attracting attention worldwide mainly due to AM resistance spreading. The role of AM prophylaxis in the modulation of gut microbiota, as well as of gut health, is still not clearly understood. Therefore, this study aimed to investigate the role of different prophylaxis protocols in the modulation of the gut barrier in broilers by applying a histopathological approach. Intestinal tissue samples were collected from a total of 240 male broilers (Ross 306), reared and treated with different AM protocols. Haematoxylin and Eosin (HE) staining and a multiple scoring system were used to evaluate the presence of lesions in ileum, cecum and colon of treated broilers. Moreover, immunohistochemistry (IHC) was performed to assess the expression of claudin-3 and ZO-1 proteins in intestinal tissues. The application of a semi-quantitative scoring system was used in IHC stained samples. HE results revealed that intestinal tissues were mainly characterized by epithelial detachment and fusion of the intestinal villi, but also by the presence of lymphocytic infiltrate in the mucosa and submucosa of AM-treated broilers. However, the IHC approach for the evaluation of claudin-3 and ZO-1 proteins showed that their expression was not affected by the different AM treatments. Nevertheless, the presence of intestinal lesions highlighted by histopathology suggests that AM treatments could harm the gut health of broilers, inducing an inflammatory response and consequent epithelial lesions. In order to clarify the role of AM treatments in the modulation of gut barrier in broilers, further studies are needed.

## Introduction

Broiler's gut health has great importance for the poultry industry (1), in fact, gastrointestinal disorders negatively affect growth performance, animal welfare and mortality (1, 2). A complex intestinal barrier, composed of intestinal microbiota, enteric epithelial cells (IEC) and mucosal immunity, is responsible for the defense of the gut (3, 4). Gut microbiota plays a pivotal role to prevent invasion and colonization by pathogenic microorganisms (4, 5). Perturbations of the intestinal environment disrupt the homeostasis and activate a cascade of physiological events, which can lead to the instauration of an inflammatory response (1). Feed efficiency and weight gain are negatively affected by an impairment of the intestinal epithelial barrier (6). A key component of the intestinal barrier is represented by the intercellular junctional complexes, which bound together adjacent IEC. Claudins, occludin, tricellulin, and junctional adhesion molecules are the main transmembrane proteins (7, 8); on the other hand, the cytoplasmic zonula occludens (ZO) proteins form the so-called intracellular plaque, that interacts with the actin filaments of the cellular cytoskeleton (8, 9). Among several isoforms of tight-junction (TJ) proteins, it has been demonstrated that claudin-1, claudin-3, claudin-5, claudin-16, ZO-1, and ZO-2 are also expressed in the chicken intestinal epithelium (7, 10). TJs regulate the paracellular pathway and form a selective barrier for the passage of ions and molecules (7). The permeability of this barrier is highly dynamic and it depends on the expression and interaction of the different TJ proteins (7, 9). Regulation of the composition and expression of TJ complexes is affected by different internal and external factors, such as inflammatory regulators (i.e., cytokines), dietary components, microorganisms or enterotoxins (7, 8, 11). The alteration of TJ can lead to the arousal of the leaky gut syndrome, characterized by increased gut permeability, loss of nutrients, water and ions (7, 12). Furthermore, several studies established that TJ proteins are involved in the pathogenesis of enteric pathogens, leading to disruption or relocation of these proteins (7, 9, 13, 14). However, intestinal inflammation is also supposed to interact and modulate TJ expression by the activation of myosin light chain kinase or Rho-associated GTPase pathway (7). In addition, recent findings revealed that antimicrobials (AMs) could interact with host tissues, modulating physiological and pathological pathways (15, 16). AMs have been used since their discovery for the treatment of bacterial infections, but the way AMs act directly with host tissues is still not clearly understood. In this study, we hypothesized that AM treatments harmed the intestinal barrier in broilers. Therefore, we investigated the effects of different AMs prophylaxis protocols on the histological morphology of ileum, cecum and colon tissues in broilers. Secondly, we examined the expression pattern of ZO-1 and claudin-3 proteins for the assessment of the TJ complexes.

## Materials and Methods

### Animals and Tissues Collection

The broiler rearing conditions were previously described by Giannuzzi et al. (17) and Cuccato et al. (18). Concisely, 240 male broilers (Ross 308) were part of a zootechnical trial and they were reared under the same conditions in the chicken broiler farm facility of the Department of Veterinary Sciences of the University of Turin during 2018. The chicken broilers were housed in the Teaching Farm of University of Turin, that had 8 pens. The farm veterinarian routinely employed several prophylaxis protocols to prevent the gastrointestinal disorders or respiratory diseases. Our study has taken into consideration the most used AM protocols adopted in poultry farming in Italy (Table 1) as follows: thiamphenicol (THP), amoxicillin (AMX), sulfadiazine + trimethoprim (TRIM), thiamphenicol + diclazuril (THP + DCZ), amoxicillin + diclazuril (AMX + DCZ) and diclazuril (DCZ). No prophylaxis protocol was applied in 1 pen, these animals (*n* = 60) were used as control (K). Each pen was prepared with fresh wood shavings as litter. Feed and water were provided *ad libitum* with a bucket-type feeder and drinker. In all pens, the environmental conditions (lighting program, temperature, relative humidity, and ventilation rates) were controlled accordingly to the Ross broiler management guidelines. The withdrawal periods were respected before slaughtering. Detailed prophylactic protocols are available in Table 1. At the end of the cycle (58 days), broilers were regularly slaughtered after regular stunning and exsanguination, and intestinal tissues (ileum, ceca and colon) were sampled from 120 animals within 1 h after death. In particular, in each bird a segment of ~3 cm were collected at ileo-caeco-colic junction level, fixed into 10% buffered formalin (pH 7.0) and paraffin-embedded according to routine histological procedures.

**Table 1:** Detailed antimicrobial (AM) protocols for the seven groups. AMs were administered via drinking water and withdrawn before slaughtering.

**Group**	**AMs**	**Prophylactic protocols**	**Periods of treatment** **(Rearing days)**	**Withdrawal (days)**
AMX	Amoxicillin	30 mg kg^−1^ BW. twice/day	20–22/53–56	1
AMX + DCZ	Amoxicillin Diclazuril	30 mg kg^−1^ BW. twice/day 1 mg/kg	20–22/53–56 0–52	1 5
THP	Thiamphenicol	65 mg kg^−1^ BW/day	23–25/47–51	6
THP + DCZ	Thiamphenicol Diclazuril	65 mg kg^−1^ BW/day 1 mg/kg	23–25/47–51 0–52	6 5
TRIM	Sulfadiazine Trimethoprim	20 mg kg^−1^ BW/day 4 mg BW/day	21–25/50–54	3
DCZ	Diclazuril	1 mg/kg	0–52	5
K	–	–	–	–

### Histopathology

Representative sections of each sample were stained with hematoxylin-eosin (HE). All slides were observed with a Nikon Eclipse E600 light microscope (Nikon Corporation, Tokyo, Japan). A multiple grading scoring system, previously adopted in the histopathological evaluation of the swine intestine by Ruggeri et al. (19), was implemented for the evaluation of the intestinal tissues of broilers. Status of the villi/epithelia, inflammatory infiltrate in the mucosa and submucosa considering the main cellular components (i.e., lymphocytes, eosinophils) and hemorrhages were the main features evaluated. Severity and distribution scores for each of the analyzed parameters were defined as mild, moderate, and severe, and as focal, disseminated and diffuse. Moreover, for intestinal epithelia and villi, a further score, reflecting lesions severity, was assigned: normal aspect, presence of epithelial detachment, fusion, and necrosis. Finally, hyperemia and cecal tonsils reactivity were recorded. Cecal tonsils were considered reactive when an evident hypertrophy of the secondary lymphoid follicles was present and/or septa of connective tissue dividing tonsillar units were not evident, due to the proliferation of the lymphoid cells. A detailed scheme of the scoring system used with all parameters evaluated and their corresponding numerical values are presented in Table 2. A total score was obtained for each of the analyzed parameter, combining each of the registered data.

**Table 2:** Histopathological scoring system used for the evaluation of intestinal tissues in broilers. Each parameter was evaluated for all chickens in ileum, cecum, and colon.

**Parameter evaluated**	**Lesion**	**Severity**	**Distribution**
Villi and epithelium	0.5 = epithelial detachment 1 = villi fusion 2 = necrosis	1 = mild 2 = moderate 3 = severe	1 = focal 2 = disseminated 3 = diffuse
Mucosal and submucosal infiltrate		1 = mild 2 = moderate 3 = severe	1 = focal 2 = disseminated 3 = diffuse
Hyperemia	+ = present – = absent		
Hemorrhages		1 = mild 2 = moderate 3 = severe	1 = focal 2 = disseminated 3 = diffuse
Cecal tonsils	+ = activated – = silent		

### Immunohistochemistry

Immunohistochemistry (IHC) was performed on six randomly selected animals of each treatment group for ileum, cecum and colon. After deparaffinization, endogenous peroxidases were blocked using a 0.3% H_2_O_2_ solution for 30 min. Antigen retrieval was achieved by incubation in Tris-EDTA buffer (pH 9.0) at 98° C for 30 min. After a 5 min washing step in phosphate-buffered saline (pH 7.4), slides were incubated with Normal Horse Serum 2.5% for 10 min in a humidifier chamber. To evaluate TJs proteins, two primary antibodies were chosen: anti-claudin 3 (rabbit polyclonal antibody specific to Claudin 3, ab15102, Abcam, Cambridge, UK) and anti-ZO-1 tight junction protein antibody (rabbit monoclonal [EPR19945-224] antibody specific for ZO-1 tight junction protein, ab221546, Abcam, Cambridge, UK). Tissue slides were incubated overnight at 4°C with primary antibodies in a humidifier chamber. Antibodies detection was performed by avidin-biotin-peroxidase complexes, using the Vectastain Universal Quick HRP Kit (Vector Laboratories, Burlingame, VT). A diaminobenzidine-hydrogen peroxide solution (DAB substrate kit, Vector Laboratories, Burlingame, VT) was used for 5 min as a chromogen. Tissue slides were washed in water, hematoxylin counterstained, dehydrated and mounted with a coverslip. Positive controls were performed to ensure the reactivity of primary antibodies and negative controls without primary antibodies were also conducted to avoid the presence of non-specific signals in chicken intestinal samples. Chicken ileal tissue was used as positive control for claudin-3 antibody and murine renal tissue for ZO-1 antibody, as suggested by the manufacturer. A quantitative scoring system was used to evaluate ZO-1 and claudin-3 expression levels (0–100%). The examination was carried out with a Nikon Eclipse E600 microscope (Nikon Corporation, Tokyo, Japan). Score assignment was performed for each sample using a 40x lens and five different areas were randomly evaluated in ileum and colon, as 10 different areas in cecum, equally divided between the two ceca.

### Statistical Analyses

Histology results were analyzed using GraphPad Prism v.8 (GraphPad Software, California, USA). Data were checked for population normality with the Kolmogorov-Smirnov test. Scores were compared among the different groups from the three gut tracts using the Kruskal-Wallis test, followed by Dunn's post-tests. For IHC, data obtained from different groups from the three intestinal tracts were analyzed by nested one-way ANOVA. *P*-value <0.05 was considered significant.

## Results

### Histopathology

**Figure 1:**
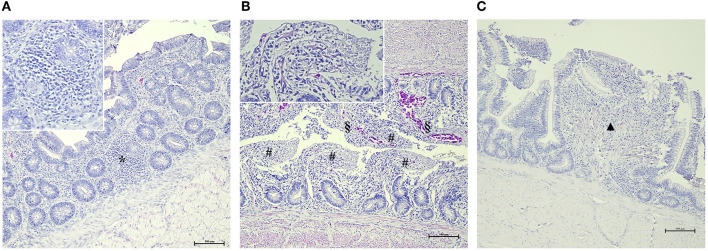
Main histological gut findings in AM-treated groups. **(A)** Focal lymphocytic infiltration (*) of the mucosa (HE, 200x – insert 400x). **(B)** Diffuse and severe epithelial detachment (#) associated with hyperemia (§) (HE, 200x – insert 400x). **(C)** Focal epithelial fusion of the villi (▲) (HE, 200x – insert 400x).

**Figure 2:**
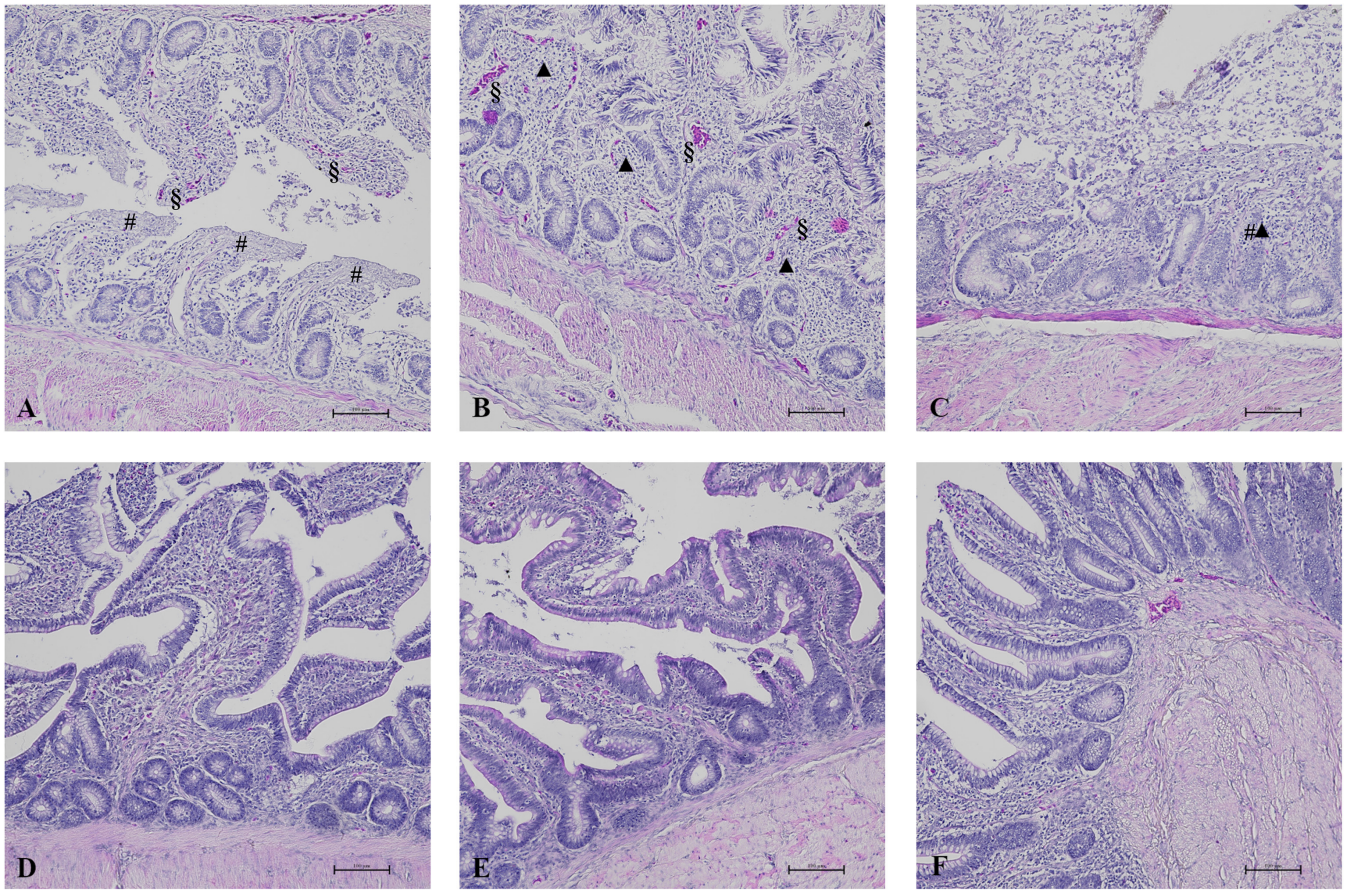
HE results in broilers of zootechnical trial. In AM-treated groups, ileum **(A)** showed diffuse and severe epithelial detachment (#) associated with hyperemia (§) (HE, 100x) and cecum **(B)** showed severe and diffuse epithelial fusion (▲) of the villi associated with focal epithelial detachment of the villi (#) and hyperemia (§) (HE, 100x). Colon **(C)** was characterized by severe and diffuse epithelial fusion of the villi (▲) associated with severe and diffuse epithelial detachment of the villi (#) (HE, 100x). These HE results were compared to the normal aspect of intestinal villi and epithelium of the respective intestinal tract observed in K group **(D–F)**.

In this study, we investigated the effect of different AM prophylaxis protocols on the gut health of treated broilers. Histopathological evaluation revealed that intestinal villi of the AM-treated groups were characterized by epithelial detachment and fusion often associated with lymphocytic infiltration (Figure 1). Moreover, several samples were characterized by hyperemia and reactivity of cecal tonsils. Histological features of the intestinal tracts analyzed are showed in Figure 2.

**Figure 3:**
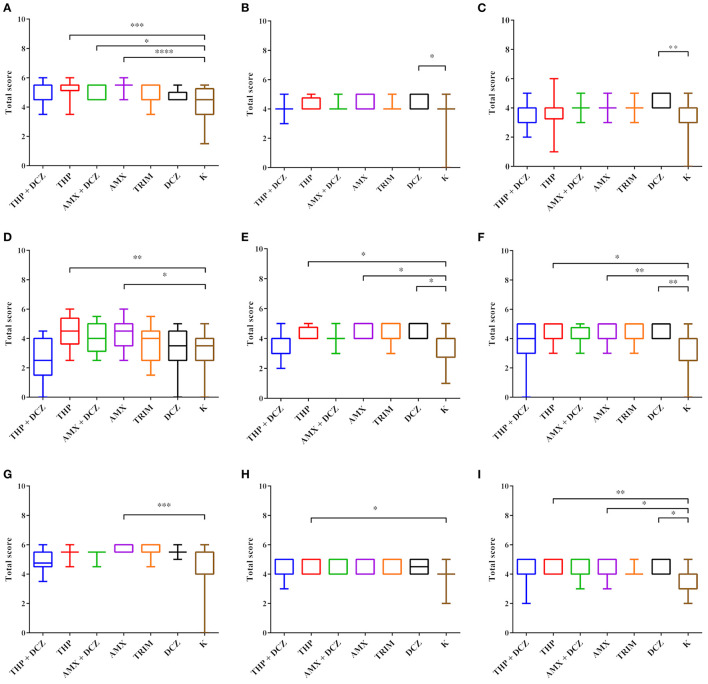
Statistical analysis of HE scores. Box plot graphs were generated for ileum **(A,D,G)**, cecum **(B,E,H)** and colon **(C,F,I)**. The data for the villi total scores **(A–C)**, the lymphocyte score in the mucosa **(B,E,H)** and the lymphocyte score in the submucosa **(C,F,I)** are, respectively, shown. Results were considered significant with *p-*value < 0.05 (*p < 0.05; **p < 0.01; ***p < 0.001; ****p < 0.0001).

A list of *p*-values generated by Kruskal Wallis analyses is presented in Table 3. Results of total scores analyses of histology evaluation (epithelial lesions and mucosa/submucosa infiltration) are presented in the box plots of Figure 3. Bars and asterisks point out significant differences revealed by post-tests. Intriguingly, THP and AMX groups most frequently showed statistically significant increased epithelial lesions and severe and diffuse lymphocytic infiltration in the mucosa and submucosa of all intestinal tracts. In addition, a significantly increased lymphocytic infiltrate was also found in the mucosa and submucosa of animals belonging to DCZ group in all the examined tracts.

**Table 3:** Summary results (*p*-value) of HE histology analyzed by Kruskal Wallis test.

**Intestinal tract**	**Epithelial lesions**	**Mucosa infiltrate**	**Submucosa infiltrate**
Ileum	<0.0001	0.0141	0.0063
Cecum	0.0002	0.0139	0.0031
Colon	0.0007	0.043	0.0064

### Immunohistochemistry

**Figure 4:**
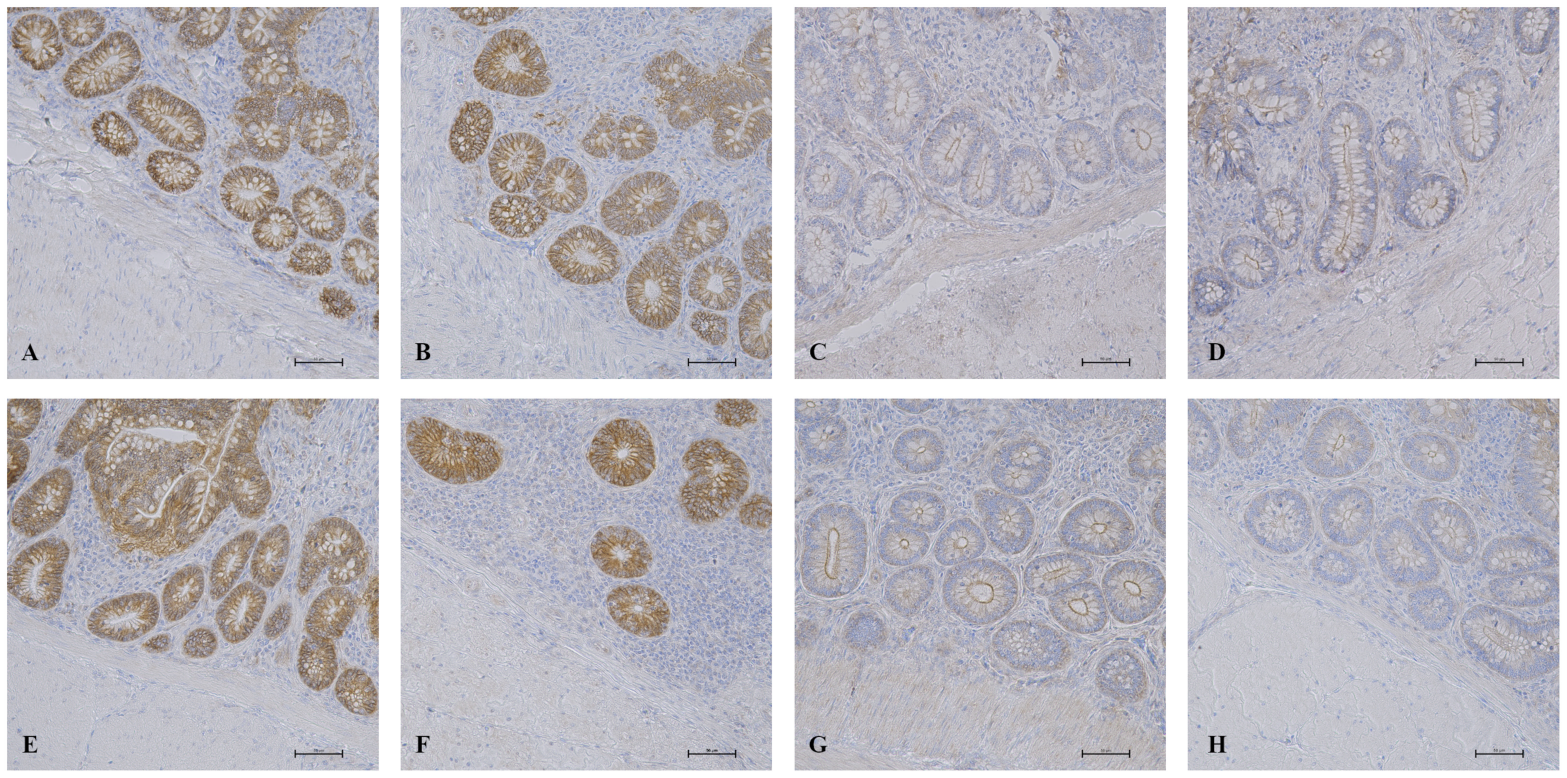
IHC results in the intestinal mucosa. Histological pictures of claudin-3 in ileum **(A)** and cecum **(B)** of AM-treated groups and in ileum **(E)** and cecum **(F)** of K group. ZO-1 expression in ileum **(C)** and cecum **(D)** of AM-treated groups and in ileum **(G)** and cecum **(H)** of K group. IHC, 200x magnification.

Results for the expression of claudin-3 and ZO-1 proteins evaluated by IHC in intestinal samples are summarized in Figure 4. No significant differences between treated groups and controls were revealed for both proteins. However, the coefficient of variation in the ANOVA test for ZO-1 protein was high for all groups (between 66.58 and 119%).

## Discussion

Gut health relies on the maintenance of the feeble balance between the host, the intestinal microbiota, the intestinal environment and dietary compounds. This balance can be significantly affected by factors such as bird management, feed quality and the environment. In this context also AMs use can lead to different negative consequences primarily for the gut microbiota (20, 21), but even more interestingly, AMs can modulate the host transcriptome in different intestinal tracts and other organs, e.g., liver and skeletal muscle (15–17). It is well-known that AM-induced dysbiosis can negatively influence host physiology and predispose to infectious diseases, but also to other disorders as well as metabolic disease or inflammation (4, 21, 22). However, few studies have focused their attention on the role of prophylactic and therapeutic doses of AM in broiler gut health. In this study, we investigated the status of the gut barrier through the application of a multiple scoring system in HE stained samples. In treated broilers, we found that the main intestinal lesions were characterized by detached or fused epithelia. Moreover, a severe and diffuse lymphocytic infiltrate was found in the mucosa and submucosa of ileum, cecum and colon of AM-treated broilers. These findings suggest that AM use in broilers has a direct impact on the gut barrier. The presence of villi damage and lymphocytic infiltrate in AM-treated animals presumes the presence of a trigger stimulus that induces the pathological process. It is widely known that specific enteric pathogens induce an inflammatory response in the broiler intestine (7, 23). However, also gut dysbiosis can induce the production of pro-inflammatory cytokines and initiate the inflammatory cascade (4, 7). All administered AM have broad-spectrum activity and the gut microbiota in ileum and cecum were negatively affected in alpha and beta metrics by AM treatments (18). It is well-known that a healthy gut microbiota is a crucial part of the intestinal barrier and thus, for the defense of the host. Normally, microbe-associated molecular patterns (MAMPs) of the gut microbiota do not induce any activation of the pattern recognition receptors and consequently do not initiate a pro-inflammatory response, keeping a controlled homeostasis of the intestine (4, 6). As already demonstrated in our previous work (18), the AM treatments induced a dysbiosis status in the ileal and cecal microbiota, that was not recovered at the end of the rearing cycle. The presence of an altered microbiota in treated broilers may have led to break the balanced homeostasis between gut microbiota and host, inducing the activation of the pro-inflammatory cascade. Moreover, these results support the hypothesis that AM effects are not only related to the gut microbiota, but directly involve the animal host and its tissues (24). Intriguingly, AMX and THP groups showed the most significant differences in all the scores analyzed. In those groups, broilers were fed without adding DCZ to the diet, but they received a coccidiosis vaccine (coarse spray; Hypracox, Amer, Spain) to ensure protection against these protozoa. The difference between groups with and without DCZ suggests a possible role of this molecule in the regulation of gut health. Moreover, statistical analysis revealed a significant increase of inflammatory infiltrate in mucosa and submucosa of DCZ treated broilers. Actually, these results were unexpected since DCZ is supposed to be active only against coccidia and other protozoa, even if the mechanism of action of DCZ is still not clearly understood (25). A recent study demonstrated that DCZ has a dose-dependent antibacterial activity against *Staphylococcus aureus, Enterococcus faecalis*, and *Streptococcus agalactiae* (26). This latest discovery suggests that probably DCZ has a broader spectrum of action than the sole anticoccidial activity. In parallel, this study wanted to evaluate the effects of AM prophylaxis on the expression of TJ proteins. Enteric pathogens, e.g. *Escherichia coli, Salmonella enterica, Campylobacter jejuni* and *Clostridium perfringens*, have the ability to target TJ components and to exploit the so-increased paracellular pathway reaching other extra-intestinal organs (7). Recently, dysbiosis has been suggested to play a role in the regulation of TJ proteins (8, 21). In our case, no statistically significant differences were shown in the expression of claudin-3 and ZO-1 proteins. Maybe, our not significant results could be due to the semi-quantitative scoring employed by pathologists on the sections stained using diaminobenzidine-hydrogen peroxide solution. It is demonstrated that in some cases TJ proteins can be regulated also by changing their location in the cells (7). Using immunofluorescence (IF), Roxas et al. (27) demonstrated that enterohemorrhagic *E. coli* infected mice showed a redistribution of claudin-3 and occludin in the enterocytes. Although our results do not confirm the abovementioned studies, a possible role in the regulation of TJ by AM-induced dysbiosis may not be excluded. Further studies are needed to better clarify the role of dysbiosis and inflammation of TJ expression, probably by IF and quantifying with a High-Content Screening System.

In conclusion, this study has associated AM prophylaxis use with the presence of histopathological lesions, while TJ proteins expression would seem unaffected by the use of AM. However, the published literature suggests that dysbiosis and inflammation can have a role in TJ regulation. In poultry farming, gut health is considered of great importance for production performance, understanding the mechanisms of gut barrier dysfunction may allow future prevention of the serious sequelae of bacterial translocation and sepsis in veterinary medicine. The morphological alterations reported in this work are additional findings, that taken together with our previously published results on zootechnical parameters, transcriptional alterations and gut microbiota (17, 18) will allow to better clarify the role of antimicrobial prophylaxes on the modulation of gut health. Thereby, further studies are needed to better understand the activity of AM against the intestinal barrier and TJ regulations.

## Data Availability Statement

The raw data supporting the conclusions of this article will be made available by the authors, without undue reservation.

## Ethics Statement

The zoo technical trial was approved by Ethics and Animal Welfare Committee of the Department of Veterinary Sciences (approved on 5th January 2018, protocol n. 275/22). (https://www.veterinaria.unito.it/do/organi.pl/Show?_id=twsn). All applicable international, national and/or institutional guidelines for care and use of animals were followed. The authors further specify that intestinal samples were collected at the end of the rearing cycle during a regular and commercial slaughter.

## Author Contributions

FC designed the study. FC, BB, SD, PP, and FS contributed to the zootechnical trial and samples collections. MC, FC, SD, CC, and FS acquired the data. PP performed the statistical analyses. MC performed the data analysis and interpretation and wrote the manuscript. FC, SD, PP, and FS revised the manuscript. All authors reviewed this manuscript. All authors contributed to the article and approved the submitted version.

## Funding

This study was supported by Ministero dell'Istruzione, dell'Università e della Ricerca (MIUR) under the program Dipartimenti di Eccellenza ex L.232/2016 to the Department of Veterinary Science, University of Turin, Italy. The funders had no role in study design, data collection and analysis, decision to publish, or preparation of the manuscript.

## Conflict of Interest

The authors declare that the research was conducted in the absence of any commercial or financial relationships that could be construed as a potential conflict of interest.

## Publisher's Note

All claims expressed in this article are solely those of the authors and do not necessarily represent those of their affiliated organizations, or those of the publisher, the editors and the reviewers. Any product that may be evaluated in this article, or claim that may be made by its manufacturer, is not guaranteed or endorsed by the publisher.
